# Randomized Controlled Pilot Study Testing Use of Smartphone Technology for Obesity Treatment

**DOI:** 10.1155/2013/151597

**Published:** 2013-12-10

**Authors:** Jerilyn K. Allen, Janna Stephens, Cheryl R. Dennison Himmelfarb, Kerry J. Stewart, Sara Hauck

**Affiliations:** ^1^Johns Hopkins University Schools of Nursing, Medicine and Public Health, 525 N. Wolfe Street, Baltimore, MD 21205, USA; ^2^Johns Hopkins University School of Nursing and Medicine, 525 N. Wolfe Street, Baltimore, MD 21205, USA; ^3^Johns Hopkins University School of Medicine and Nursing, 301 Building Suite 2422, 4940 Eastern Avenue, Baltimore, MD 21224, USA; ^4^Institute for Clinical and Translational Research, Johns Hopkins University School of Medicine, 1820 Lancaster Street Suite 300, Baltimore, MD 21231, USA

## Abstract

*Background*. The established interventions for weight loss are resource intensive which can create barriers for full participation and ultimate translation. The major goal of this pilot study was to evaluate the feasibility, acceptability, and preliminary efficacy of theoretically based behavioral interventions delivered by smartphone technology. *Methods*. The study randomized 68 obese adults to receive one of four interventions for six months: (1) intensive counseling intervention, (2) intensive counseling plus smartphone intervention, (3) a less intensive counseling plus smartphone intervention, and (4) smartphone intervention only. The outcome measures of weight, BMI, waist circumference, and self-reported dietary intake and physical activity were assessed at baseline and six months. *Results*. The sample was 78% female and 49% African American, with an average age of 45 years, and average BMI of 34.3 kg/m^2^. There were trends for differences in weight loss among the four intervention groups. Participants in the intensive counseling plus self-monitoring smartphone group and less intensive counseling plus self-monitoring smartphone group tended to lose more weight than other groups (5.4 kg and 3.3 kg, resp.). *Conclusions*. The results of this pilot trial of a weight loss intervention provide preliminary support for using a smartphone application for self-monitoring as an adjunct to behavioral counseling.

## 1. Introduction

More than one-third of US adults (35.7%) are obese [1] which greatly increases their risks for hypertension, hyperlipidemia, type 2 diabetes, heart disease, stroke, and some types of cancer. Even modest weight loss of 5%–10% of initial body weight can reduce the risk of these negative health consequences [2]. National guidelines target the reduction of total and abdominal obesity through increased physical activity and caloric restriction [3]. Although research has demonstrated the efficacy of these lifestyle changes on weight loss and improvement of cardiovascular risk factors, promotion and maintenance of such changes continues to be a challenge [4–6]. The established interventions are resource intensive and require frequent group and individual in-person counseling sessions which can create barriers for full participation and ultimate translation. Busy health professionals need effective tools and strategies to facilitate healthy eating and increase physical activity in their patients, especially those who are overweight or obese.

Communication technologies such as smartphones offer a potentially powerful approach for addressing common barriers to health behavior change through delivering convenient, individually tailored, and contextually meaningful behavioral interventions. There is research evidence suggesting that mobile phones are a useful tool for interventions seeking to improve health outcomes [7, 8]. However, rigorous clinical trials testing state-of-the-art technologies applying strong theoretical models while isolating the effect of technology are limited.

Although there are close to 6000 consumer health applications for smartphones, few applications have been subjected to clinical trials to test effectiveness in changing health behaviors.

The major goals of this pilot study were to evaluate the feasibility, acceptability, and preliminary efficacy of theoretically based behavioral interventions delivered by smartphone technology to increase physical activity and decrease caloric intake resulting in weight loss and improvements in body composition. An additional goal was to assess trends in differences in effectiveness among the interventions, the recruitment and screening yield, adherence and retention rates, and the acceptance of the technology.

The SLIM (Smart coach for LIfestyle Management) study randomized 68 eligible participants to receive one of four interventions for six months: (1) an established intensive diet and exercise counseling intervention, or (2) an established intensive diet and exercise counseling plus self-monitoring smartphone intervention, or (3) a less intensive diet and exercise counseling plus self-monitoring smartphone intervention, or (4) self-monitoring smartphone intervention only. We hypothesized that participants randomized to the counseling plus self-monitoring smartphone technology would achieve greater weight loss than those in the counseling or smartphone only groups. We further hypothesized that we could accomplish similar results with a more translatable and potentially more cost-effective less intensive in-person intervention when augmented by the self-monitoring smartphone technology.

## 2. Methods

### 2.1. Participants

Participants were recruited through a variety of strategies used successfully in our other studies such as flyers, physician referrals, and existing lists of volunteers from prior studies of the investigators. Individuals between 21 and 65 years of age with body mass index (BMI) of 28–42 kg/m^2^ who had an iPhone or Android phone and were willing to download the application to be used on their devices were eligible to participate. Individuals were excluded if they had a history of myocardial infarction, angina, coronary artery bypass graft surgery, percutaneous transluminal coronary angioplasty, congestive heart failure, or diabetes. They could not have conditions significantly limiting exercise such as active cancer treatment, peripheral arterial disease, severe orthopedic problems, or pain limiting arthritis. They were excluded if they were currently participating in another structured weight loss program, were pregnant or planned to become pregnant in the next six months, were taking weight-loss medications, or reported a history of psychiatric illness, alcohol, or substance abuse within the past 12 months. All participants provided written informed consent. The protocol was approved by the Johns Hopkins University Institutional Review Board.

### 2.2. Outcome Measures

Data on the outcome and lifestyle behaviors were collected at the time of randomization and at 6 months. Weight and height were measured with research participants in light clothing using a stadiometer and balance scale. Body mass index was calculated as weight in kilograms/height in meters squared. Waist circumference was measured with a laminated measuring tape according to the obesity guidelines [2].

Physical activity was evaluated with the Stanford 7-Day Physical Activity Recall. This interviewer administrated survey estimated total daily energy expenditure by asking research participants to report the number of hours spent in sleep and activities classified into moderate, hard, and very hard activities over the previous seven days [9, 10]. Light activity was calculated as the remaining time. Average daily time spent in moderate or greater activity was determined. Dietary intake data were collected from 3-day food records and analyzed using the Nutrition Data System for Research (NDSR) software version 2012, Nutrition Coordinating Center (NCC) at the University of Minnesota, Minneapolis, MN.

### 2.3. Process Measures

The process evaluation was completed using several metrics. We determined the yield of recruitment strategies, reasons for exclusion, retention rates, and attendance at counseling sessions. In the counseling plus smartphone groups, we monitored the average number of entries per week for diet and exercise to observe patterns of use. In addition, we did in-depth interviews with participants as they completed the study to determine acceptability and satisfaction with the intervention. Questions also focused on the timing, quality, and impact of the program. We asked about the ease of use and acceptability of the smartphone technology in the relevant groups.

### 2.4. Interventions

The behavioral interventions were based on an eclectic theoretical approach using multiple behavioral theories: social cognitive theory, behavioral self-management, and motivational interviewing counseling techniques that were used in our prior studies [5, 6, 11]. Goals for 5% weight loss and at least 150 minutes of moderate or greater intensity physical activity were the same in all groups. The intensity of counseling sessions, defined as the frequency of in-person contact, varied between groups. Participants in the more intensive intervention groups received healthy eating and exercise counseling from a nutritionist coach weekly for the first month and biweekly for the second through sixth month. Participants in the less intensive counseling plus smartphone intervention received healthy eating and exercise counseling from the nutritionist twice during the first month and then monthly from two to six months. In-person nutritional counseling focused on decreasing calories and the DASH dietary recommendations of increasing fruits and vegetables, whole grains, and low-fat dairy products while limiting total fat, saturated fat, and dietary cholesterol. The goal for exercise was 150 minutes of moderate or greater intensity physical activity per week. The counseling sessions were one hour in length. Participants in the smartphone only group received one session of basic nutrition counseling and training in the smartphone application.

The Lose It! weight loss application promoted self-management and mindful empowerment and provided real-time feedback and motivators and opportunities for social networking and support. To activate the system, the participants entered their baseline weight, target weight, height, gender, and age. The system used the Mifflin equation for calculating resting metabolic rate along with a standard activity factor and entered target weight to establish the daily calorie budget.

The participant recorded food intake and exercise using a simple touch screen. Instant, real-time calculation of current energy balance allowed the participant to keep on track for the day and helpful charts and graphs tracked progress. Participants also were encouraged to weigh themselves weekly and record the weight in the application.

### 2.5. Statistical Analysis

Group differences in baseline sociodemographic and anthropometric characteristics were examined using ANOVA and chi-square tests. A similar analysis was completed looking for differences between study completers and those who did not complete the six-month followup. The primary outcomes were changes from baseline to six months in weight in kilograms and percentage reduction in weight, BMI, and waist circumference. Secondary outcomes included changes in diet and physical activity. Outcome data were analyzed using the nonparametric Wilcoxon signed rank test. Due to the uneven and relatively high attrition rates (31%–41%) among the four groups, we chose not to impute data or carry forward the baseline value for missing data for an intention-to-treat analysis. However, a sensitivity analysis imputing data, carrying the last observation forward and analysis only on those who completed the six-month followup, did not produce different results. Given that this was an exploratory pilot study, we were not powered to detect statistically significant differences between the groups. Statistical analyses were carried out using STATA Data Analysis and Statistical Software, version 12.

## 3. Results

### 3.1. Baseline Characteristics

Baseline characteristics of participants by group are shown in Table 1. Of the 68 participants enrolled, 78% were female and 49% were Black. The overall average age was 45 ± 11 years and BMI 34.3 ± 3.9 kg/m^2^. A majority were college educated (68%), married (57%), and employed full-time (84%). There were no significant differences in sociodemographic and baseline anthropometric measures among the intervention groups.

### 3.2. Recruitment and Retention

**Figure 1:**
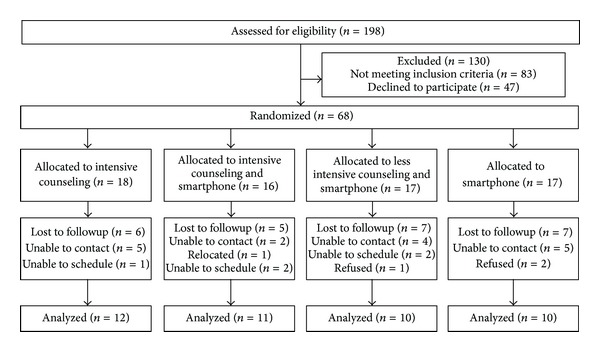
Study flow diagram.

Figure 1 is the CONSORT diagram reporting the participant flow through the study. We assessed 198 volunteers for eligibility. The largest proportion of those expressing interest in the study was recruited from physician office posters/flyers and direct referrals from our primary care physicians network (39%), existing lists of volunteers from prior studies of the investigators (28%), and friends and family of participants (12%).

A total of 110 volunteers (56%) met the eligibility criteria. Of those who were qualified to participate, 42 declined participation. The primary reason for refusal was the inconvenient time for study visits. A total of 68 individuals were randomized to one of the four groups, which represented 34% of those who originally expressed interest in participating and 62% of those who met the eligibility criteria and were invited to participate.

Forty-three (63%) returned at six months for follow-up measurements. Retention rates in the four groups ranged from 59% to 69%. There were no significant differences on baseline characteristics between those who completed the six-month evaluation compared with those who dropped out. In addition, there were no differences in dropout based on sex or ethnicity.

### 3.3. Utilization of Interventions

Adherence to the recommended intervention varied across groups (Table 2). Utilization was calculated with a ratio of the number of counseling sessions or actual days of logging relative to the possible number of sessions or days. Intervention usage was the highest overall in the intensive counseling plus smartphone group, where participants attended an average of 72% of the 14 counseling sessions and logged their diet an average of 53% and physical activity 32% of possible days in their smartphone application. The percentages for the less intensive counseling and smartphone group were very similar.

### 3.4. Satisfaction

When asked in an open-ended question what they liked most about the SLIM program, the four most prevalent responses were accountability and structure (28%), smartphone application (25%), counseling sessions (23%), and the combination of counseling sessions and smartphone application use (12%). In a similar open-ended question soliciting possible changes to improve the SLIM program, 23% suggested a stronger emphasis on exercise, 21% wanted additional feedback, and 21% recommended no changes. All participants (100%) agreed that an exercise tracking device would provide useful feedback and motivation to increase physical activity. A large majority (76%) agreed that a wireless scale that synchronized weight to their smartphone application would be motivating.

### 3.5. Change in Anthropometric and Process Measures

Table 3 compares the changes in anthropometric and process measures after the six-month intervention. There were nonsignificant trends for differences in weight loss among the four intervention groups. Participants in the intensive counseling plus self-monitoring smartphone group and less intensive counseling plus self-monitoring smartphone group tended to lose more weight than other groups. Participants in the self-monitoring smartphone group lost the least weight. However, given the small sample size and the pilot nature of the study, the effects were not statistically significant between the four groups. Similar trends were observed in changes in waist circumference, BMI, and percent weight loss. Of those who completed the six-month followup, 64% of participants in the intensive counseling plus self-monitoring smartphone group and 40% in the less intensive counseling plus self-monitoring smartphone group achieved greater than or equal to 5% decrease in their body weight. In contrast, only 25% in the counseling only group and 20% in the self-monitoring smartphone only group achieved at least a 5% weight loss. There were no differences in weight loss based on age; however, females were more likely to lose weight compared to males (*P* = 0.005) (Data are not shown.)

Self-reported physical activity of moderate or greater intensity appeared to decrease in all groups except for a slight increase in the smartphone only group. Three-day food records showed a decrease in total kilocalorie consumption, percentage of calories from fat, and dietary intake of sodium across all groups. The average number of servings of fruits and vegetables increased in all groups except the smartphone only group who reported a slight decrease.

## 4. Discussion

This pilot trial has shown the combination of personalized counseling and self-monitoring by smartphone to be a feasible and acceptable weight loss intervention. Although not powered to detect statistically significant between group differences in changes in weight, the trends supported our a priori hypothesis that participants randomized to the counseling plus self-monitoring smartphone technology would achieve greater weight loss than those in the counseling or smartphone only groups. Mean weight loss at 6 months in the intensive counseling plus self-monitoring smartphone group, − 5.4 kg (4.0), was clinically significant and comparable to weight loss achieved at six months in the POWER Trial. At 6 months in the POWER Trial, the mean (±SE) adjusted change in weight from baseline was − 1.4 ± 0.4 kg in the control group, − 6.1 ± 0.5 kg in the group receiving remote support only, and − 5.8 ± 0.6 kg in the group receiving in-person support [12]. There was no clinically meaningful weight loss and substantial dropout in the group who received no weight loss counseling which supports the need for some degree of personal contact and coaching beyond self-monitoring alone.

There also was some support for our hypothesis that we could accomplish similar results with a more scalable and potentially more cost-effective, less intensive, in-person intervention when augmented by the self-monitoring smartphone technology. Participants in the less intensive counseling plus self-monitoring smartphone group lost an average of 2.1 kg less weight as compared to the intensive counseling plus self-monitoring smartphone group. It is questionable whether this is a clinically meaningful difference in weight loss.

Secondary outcomes provide feedback on changes in lifestyle behaviors such as dietary choices. Use of the self-monitoring smartphone technology, regardless of intensity of counseling, showed to be more effective at decreasing participants' percent intake of calories from fat. At baseline, participants on average consumed slightly above the recommended macronutrient distribution range (AMDR) of 20–35% of calories from fat as recommended by the Institute of Medicine [13]. After the six-month intervention, the groups utilizing the self-monitoring smartphone technology showed a greater decrease in the percent of calories from fat than the intensive counseling alone, and the percentage of calories from fat also fell within the AMDR and approached meeting the goals of the DASH diet of <25% calories from fat. Contrasting with percent calories from fat, greater reduction of sodium intake was seen with participants who had face-to-face counseling compared to those using the smartphone only, though the greatest reductions in sodium intake were seen in the intensive and less intensive counseling plus smartphone groups.

These two secondary outcomes suggest a complementary or additive effect of the face-to-face counseling and the smartphone application Lose It! displays the macronutrient distribution of a user's intake prominently on the “My Day” tab, but the user must navigate to another touch screen to obtain information on sodium intake. A trained nutrition counselor or health coach can easily identify foods high in sodium in a participant's diet log and provide education and counseling on reduction strategies during face-to-face visits. However, it may be more difficult for a nutritionist or health coach to estimate the percent calories from fat. This may explain the observed differences between groups for the percent of calories from fat and sodium intake measures.

Fruit and vegetable intake showed little improvement in 3 of the 4 groups, where increase in the number of servings of fruits and vegetables consumed ranged from 0.05 to 0.81 servings. Only the less intensive counseling group plus self-monitoring smartphone technology showed a large increase in the number of fruits and vegetables consumed with an additional 2.1 servings per day, though they had the lowest number of servings of fruits and vegetables at baseline.

This pilot study has several strengths. Although not powered to detect statistically significant differences in weight change between groups, the randomized design equalized groups on important baseline characteristics and allowed investigators to observe trends in effectiveness of the four interventions. In addition, it was designed to examine the effect of decreasing face-to-face counseling sessions supplementing with smartphone technology for self-monitoring which could be a cost-effective and translatable strategy. The self-monitoring smartphone application is a popular commercially available application which has not been comprehensively evaluated.

Study limitations included an overall attrition rate of 37% and the attrition was not equal among the groups. Generalizability of the pilot study results is limited given that the sample was predominately female. However, it is noteworthy that 49% of the participants were African American. Twenty-eight percent of those who completed the trial also reported that at some time during the trial they had used another weight loss intervention (e.g., computer programs or smartphone applications) in addition to their originally allocated intervention.

## 5. Conclusions

This pilot trial of a weight loss intervention using a smartphone application for self-monitoring as an adjunct to behavioral counseling has provided valuable data to inform a larger randomized controlled trial. Given the attrition rates and unequal dropout, a larger trial will need to implement robust retention strategies. Two control groups (IC and SP only groups) and two levels of intensity of counseling with smartphone self-monitoring groups were used in this pilot. It may be more cost-effective for a full trial to compare counseling plus smartphone self-monitoring, the seemingly most robust intervention, with what is currently offered as standard of care for weight loss in primary care settings. Testing in a larger trial, a state-of-the-art mobile technology application that represents a convenient and increasingly available and acceptable means of reaching a substantial proportion of the population has the potential to provide evidence to support an intervention that could impact substantially the serious public health problem of obesity.

## Figures and Tables

**Table 1:** Baseline sample characteristics by treatment group.

Characteristics	Total (*n* = 68)	IC (*n* = 18)	IC + SP (*n* = 16)	LIC + SP (*n* = 17)	SP (*n* = 17)	*P* value
Age, years, mean (SD)	44.9 (11.1)	42.5 (12.1)	45.6 (9.3)	46.4 (9.6)	45.3 (13.2)	0.74
Weight, kg, mean (SD)	97.3 (16.2)	96.0 (17.4)	100.3 (16.5)	96.8 (14.8)	96.4 (16.9)	0.78
BMI, kg/m^2^, mean (SD)	34.3 (3.9)	34.1 (4.1)	34.3 (3.9)	33.5 (3.5)	35.3 (4.1)	0.70
Male waist circumference cm, mean (SD)	117.3 (11.6)	117.3 (15.5)	119.4 (11.6)	116.4 (4.6)	113.8 (23.0)	0.91
Female waist circumference cm, mean (SD)	107.4 (11.4)	106.4 (14.5)	109.7 (11.4)	108.7 (8.4)	105.5 (11.1)	0.47
Female, *n* (%)	53 (77.9)	14 (77.8)	11 (68.8)	13 (76.5)	15 (88.2)	0.64
African American, *n* (%)	33 (48.5)	13 (72.2)	6 (37.5)	7 (41.2)	7 (41.2)	0.18
College educated, *n* (%)	46 (67.6)	8 (44.4)	13 (81.3)	13 (76.5)	12 (70.6)	0.29
Married, *n* (%)	39 (57.4)	10 (55.6)	10 (62.5)	10 (52.9)	9 (52.9)	0.74

IC: intensive counseling.

SP: smartphone.

LIC: less intensive counseling.

BMI: body mass index.

**Table 2:** Utilization of interventions.

Intervention use	IC (*n* = 18)	IC + SP (*n* = 16)	LIC + SP (*n* = 17)	SP (*n* = 17)
Counseling sessions attended, mean % (SD)*	58 (37)	72 (31)	66 (34)	N/A
Days of diet SP entries, median % (IQR)**	N/A	53 (37)	58 (58)	23 (39)
Days of physical activity SP entries, median % (IQR)**	N/A	32 (43)	23 (42)	9 (33)

*Rate of actual number of counseling sessions attended relative to possible number.

**Ratio of actual days of logging relative to possible number of days.

IC: intensive counseling; SP: smartphone; LIC: less intensive counseling; SD: standard deviation; IQR: interquartile range.

**Table 3:** Changes in outcomes by group.

Outcome	IC *n* = 18	IC + SP *n* = 16	LIC + SP *n* = 17	SP *n* = 17	*P* value
Body weight, kg, mean (SD)					0.89
Baseline	96.0 (17.4)	100.3 (16.5)	96.8 (14.8)	96.4 (16.9)	
Change	−2.5 (4.1)	−5.4 (4.0)	−3.3 (5.9)	−1.8 (3.7)	
BMI, kg/m^2^, mean (SD)					0.79
Baseline	34.1 (4.1)	34.3 (3.9)	33.5 (3.5)	35.3 (4.1)	
Change	−0.8 (1.4)	−1.8 (1.3)	−1.1 (2.0)	−0.7 (1.3)	
Male waist circumference, cm, mean (SD)					0.36
Baseline	117.3 (15.5)	119.4 (11.6)	116.4 (4.6)	113.8 (23.0)	
Change	−3.0 (2.4)	−7.01 (2.6)	−6.5 (0.35)	−3.38 (8.3)	
Female waist circumference, cm, mean (SD)					0.22
Baseline	106.4 (14.5)	109.7 (11.4)	108.7 (8.4)	105.5 (11.1)	
Change	−3.19 (7.4)	−5.68 (3.7)	−3.64 (7.9)	−0.88 (2.9)	
Self-reported activity ≥ moderate intensity, hrs/week, mean (SD)					0.51
Baseline	5.0 (5.2)	4.9 (5.7)	5.3 (5.4)	3.5 (3.7)	
Change	−1.4 (7.1)	−2.0 (5.4)	−3.6 (5.5)	0.19 (5.1)	
Dietary intake, kcal/day, mean (SD)					0.66
Baseline	2069.6 (463.2)	2085.7 (640.8)	1988.3 (722.7)	1647.3 (460.4)	
Change	−415.6 (376.4)	−468.2 (634.0)	−218.5 (859.5)	−249.2 (770.5)	
Calories from fat, %, mean (SD)					0.37
Baseline	36.6 (5.22)	36.2 (6.64)	36.2 (4.2)	34.5 (5.97)	
6 months	−0.67 (4.5)	−4.89 (9.3)	−4.6 (4.5)	−3.48 (12.5)	
Fruit and vegetable intake, servings per day, mean (SD)					0.61
Baseline	4.98 (2.6)	4.32 (1.5)	4.16 (2.3)	4.22 (2.1)	
Change	0.81 (2.8)	0.51 (3.2)	2.1 (3.4)	0.05 (4.9)	
Sodium intake, mg/day, mean (SD)					0.88
Baseline	3422.1 (938.1)	3665.1 (1108.9)	3645.8 (1519.7)	2842.8 (1136.8)	
Change	−517.2 (806.8)	−788.2 (1165.1)	−622.5 (1376.3)	−157.5 (2145.0)	

IC: intensive counseling; SP: smartphone; LIC: less intensive counseling; BMI: body mass index. Change values were calculated for completers at 6 months.
